# Supporting coach-led dual career guidance for student-athletes: validation of the web-based Japanese version of the dual career competency questionnaire for athletes

**DOI:** 10.3389/fspor.2025.1645690

**Published:** 2025-11-26

**Authors:** Goichi Hagiwara, Kayoko Kurita, Tadao Isaka, Jim Ueda, Katsuhiko Ishikawa, Daisuke Akiyama

**Affiliations:** 1 Department of Sport Science and Health, Kyushu Sangyo University, Fukuoka, Japan; 2 Center for Research and Development in Higher Education, The University of Tokyo, Tokyo, Japan; 3 Faculty of Sport and Health Science, Ritsumeikan University, Shiga, Japan; 4 Faculty of Sociology, Otemon Gakuin University, Osaka, Japan; 5 Graduate School of Education, Naruto University of Education, Tokushima, Japan

**Keywords:** dual career competencies, student-athletes, web-based assessment, coach support, questionnaire validation

## Abstract

**Introduction:**

The concept of a dual career, which emphasizes the integration of athletic and academic pursuits, has become increasingly central to athlete development policies worldwide. Although the Dual Career Competency Questionnaire for Athletes (DCCQ-A) has been psychometrically validated in multiple European contexts, Japan still lacks a culturally adapted and scalable tool to assess student-athletes' dual-career competencies. Therefore, this study aimed to address this gap by developing and validating a web-based Japanese version of the DCCQ-A (DCCQ-AJ) to facilitate empirical research and provide evidence-based educational interventions.

**Methods:**

Data were collected via an online survey from 1,035 university student-athletes (*M* = 19.82, *SD* = 1.12). The original 29-item DCCQ-A was translated following a back-translation protocol and evaluated across two dimensions perceived importance and perceived possession of competencies spanning four theoretical domains. Content validity was assessed using content validity coefficients (CVC); item discrimination was evaluated via good–poor (G–P) analysis. Confirmatory factor analyses (CFA) were conducted to verify the factorial structure. Internal consistency was examined using Cronbach's alpha, and paired-sample t-tests were used to assess discrepancies between perceived importance and possession levels.

**Results:**

All items demonstrated strong content validity, and G–P analysis indicated significant item discrimination (*p* < .001). CFA confirmed the four-factor structure with good model fit indices. Internal consistency was acceptable to excellent across subscales. Descriptive statistics indicated that perceived importance scores (e.g., *M* = 3.95–4.08) consistently exceeded perceived possession scores (e.g., *M* = 3.62–3.99). Paired-sample *t*-tests revealed statistically significant differences across all domains (*t* = 5.38–15.01, *p* < .001), with small-to-moderate effect sizes (Cohen's *d* = 0.17–0.47). These results underscore meaningful gaps between what student-athletes value and what they perceive themselves as possessing, indicating domains in need of targeted support.

**Conclusion:**

The DCCQ-AJ is a psychometrically sound web-based instrument for assessing dual-career competencies among Japanese student-athletes. Its scalability and structural equivalence to the original DCCQ-A render it suitable for cross-cultural comparative studies and longitudinal monitoring. This tool has practical utility for researchers and institutions aimed at evaluating and enhancing dual-career support frameworks in higher education and elite sports contexts.

## 1 Introduction

The concept of “dual career” was formally introduced in the European Commission's (EC) 2007 White Paper on Sport; its definition has since been shaped predominantly within the European context (1). According to the 2012 EU Guidelines on Dual Careers of Athletes: Recommended Policy Actions in Support of Dual Careers in High-Performance Sport, a dual career refers to “the requirements for athletes to successfully initiate, develop and finalize an elite sporting career as part of a lifelong career, in combination with the pursuit of education and/or work” (2). Within this framework, dual careers in university sports are understood as “the combination of elite sport and education,” emphasizing the simultaneous pursuit of athletic and academic development (3).

Due to the recent expansion of university admission pathways that do not require academic testing, the number of student-athletes in Japan has increased substantially (4). However, this growth has brought several challenges, including long training hours, frequent class absences due to competitions, limited time for career preparation, and inadequate academic engagement among some student-athletes (5, 6). In addition to these academic and scheduling constraints, recent international studies have highlighted systemic barriers such as insufficient flexibility in university curricula (7), limited financial aid and institutional support (8), restricted access to appropriate sports facilities (7), and sleep-related difficulties among student-athletes managing dual demands (9).These issues have raised concerns about how student-athletes can effectively balance academic responsibilities and athletic commitments within the university setting.

In response to these emerging challenges, the Japan Sports Agency established the Japan Association for University Athletics and Sports (UNIVAS) in 2019 as a national framework to support student-athletes. UNIVAS aims to promote university sports, strengthen interuniversity cooperation, enforce compliance, and enhance dual-career development through educational support and research initiatives (10). As of April 2025, it comprises 225 member universities and supports approximately 150,000 student-athletes (11).

Within its mission, UNIVAS defines a dual career as “engaging in athletic activities while concurrently participating in academic or other non-sport activities” (12). The organization emphasizes that university life offers opportunities not only to pursue specialized knowledge but also to develop leadership and personal competencies that can be applied to future societal roles. Despite these national efforts, a 2022 web-based survey (*n* = 1,694) found that 61.0% of student-athletes were unfamiliar with the concept of a dual career, and 43.1% had never considered their post-athletic career (13), indicating persistently low awareness and limited engagement in dual-career planning.

Although interest in dual careers has grown since the formation of UNIVAS, Japanese research has primarily focused on perceptions and awareness rather than specific competencies. In contrast, European countries have advanced the development of quantitative assessment tools to evaluate dual-career competencies, which are essential for understanding and supporting student-athletes.

Dual-career competencies refer to the skills and resources that enable student-athletes to balance academic demands, athletic performance, and career development. These may include self-management, career planning, emotional regulation, social support, and adaptability (14–16). Research has shown that these competencies vary depending on the athlete's developmental stage (16). Stambulova's (17) athletic career transition model and Wylleman and Rosier's (18) holistic career model highlight the evolving demands placed on athletes during sports, education, and social transitions.

By integrating these models, researchers have identified the competencies required by athletes to navigate dual career demands (19–21). Most of the research has been qualitative. For instance, Aquilina (19) interviewed elite and professional student-athletes from France, Finland, and the UK, revealing that success in both sports and education was equally important. The participants also highlighted the need for supportive networks and the ability to seek help. Tekavc et al. (21) interviewed 12 former elite athletes and found that achieving a dual career requires resilience and competencies that are transferable beyond sports. Quantitative approaches have recently emerged. Lupo et al. (21) used the Student-Athletes' Motivation towards Sports and Academics Questionnaire (SAMSAQ) to assess motivation, while Sandstedt et al. (22) applied the Student-Athlete Career Situation Inventory (SACSI) to measure career attitudes. Although useful, these tools do not capture the full range of dual-career competencies as highlighted in qualitative studies (16).

To fill this gap, the Erasmus + Sport project developed the Dual Career Competency Questionnaire for Athletes (DCCQ-A), which assesses both perceived importance and possession of dual career competencies (16, 23). The DCCQ-A consists of four dimensions: (i) dual-career management, (ii) career planning, (iii) emotional awareness, and (iv) social intelligence and adaptability. De Brandt et al. (16) found that while athletes recognized the importance of these competencies, they often lacked confidence in their abilities. Since then, DCCQ-A has been adapted for use in multiple languages. Grubertt et al. (24) validated a Portuguese version in Brazil, recommending further validation based on competitive level and developmental stage. Our research team conducted a preliminary validation of the Japanese version of the DCCQ-A. However, it lacks sufficient psychometric properties, likely due to the sample size and institutional limitations (25). Despite growing interest, Japan lacks a standardized and reliable tool for assessing dual-career competencies. Therefore, a web-based tool is required to facilitate large-scale research across diverse regions. Online assessments offer flexibility and accessibility and can serve as practical tools for coaches and managers (26, 27). Moreover, these tools enable cross-cultural comparisons, helping institutions identify and address specific support needs. Therefore, this study aimed to develop a web-based Japanese version of the DCCQ-A (DCCQ-AJ) that maintains its original structure and item count. The tool was designed to visualize student-athletes' dual-career competencies, thereby supporting coaches and administrators in identifying individual needs and informing them of the design of tailored developmental programs. Based on previous European findings, we hypothesized that student-athletes would rate the perceived importance of dual-career competencies higher than their perceived possession across all competency domains. In Japan, where student-athletes vary widely in their academic and athletic backgrounds, standardized and scalable tools offer a pathway for evidence-based, strategic support.

## 2 Methods

### 2.1 Participants

This study targeted 2,000 student-athletes enrolled in 12 universities who agreed to participate. Also, participants were required to be (a) currently enrolled university student-athletes, (b) aged between 18 and 24 years, (c) active members of official university-recognized sports clubs, and (d) engaged in competitive activities. Sampling was conducted purposively via university club coaches or staff who distributed the survey link. Responses with more than 10% missing data or implausible patterns (e.g., identical responses across all items) were excluded. After excluding incomplete or invalid responses, 1,035 participants (236 females, 792 males, and seven unspecified; *M* = 19.82, *SD* = 1.12) were included in the analysis, resulting in a valid response rate of 51.8%. Among them, 760 were engaged in Olympic sports and 275 were engaged in non-Olympic sports.

The questionnaire was administered using Google Forms (Google LLC), a secure web-based platform. Access to the survey was provided via a unique link distributed by coaches or administrative staff. Responses were encrypted during transmission and stored on Google's cloud servers, accessible only to the research team through a password-protected institutional account. No personally identifiable information was collected, and all data were anonymized before analysis in accordance with institutional guidelines. In line with the requirements of the institutional ethics committee, the anonymized data will be retained securely for a period of ten years before being permanently deleted. The platform's terms of use and privacy policy comply with major international data protection standards.

The responses were collected between December 2022 and February 2023, typically during scheduled club meetings. Prior to participation, respondents were shown an introductory page outlining the purpose of the study, the statistical anonymity of the data, and their intention to disseminate the findings. Consent to participate was obtained via a checkbox on the interface, and only those who agreed participated in the survey. This study was approved by the Research Ethics Committee of the University of Tokyo, Japan. (Approval No. 22-2, Date: May 9, 2022).

### 2.2 Survey instrument

To evaluate the reliability and validity of the Japanese version of the Dual Career Competency Questionnaire for Athletes (DCCQ-A), we used a back-translated version of the original scale (16), which had previously been translated by our research team (25). In the original DCCQ-A (16), exploratory structural equation modeling identified a 29-item, 4-factor model as the best fit, with acceptable fit indices [*χ*^2^(296) = 2,699, RMSEA = .049, CFI = .952, TLI = .934] and internal consistency (overall *α* = .91, subscale *α* = .75–.87). Based on these results, we adopted the 29 items that demonstrated the strongest model fit in prior validation studies (Table 1).

**Table 1:** English and Japanese items of the DCCQ-A.

Factors	Item code	English	Japanese
Dual career management (DCM)	DCM1	Self-discipline to manage the demands of your study and sport	学業とスポーツを両立させるための努力している
	DCM2	Ability to use your time efficiently	自分の時間を効果的に使える
	DCM3	Ability to plan conscientiously in advance	予めしっかりと計画を立てることができる
	DCM4	Dedication to succeed in both sport and study	スポーツと学業の両方での成功に注力している
	DCM5	Ability to prioritize what needs to be done	物事に優先順位をつけることができる
	DCM6	Willingness to make sacrifices and choices to succeed in sport and study	スポーツと学業において成功するために、多少の犠牲や決断は厭わない
	DCM7	Ability to make your own responsible choices with regard to your study and sport career	自分の学業とスポーツのキャリアに責任を持って決断できる
	DCM8	Ability to create individualized routines (for sport and study)	スポーツと学業に取り組むために、自分流のルーティンを作り出せる
	DCM9	Clear understanding of what it takes to succeed in sport and study	スポーツと学業で成功するために何が必要か明確に認識している
	DCM10	Belief that study and sport can positively complement each other	学業とスポーツを両方やると、両方を補い合って良くなると思う
Career planning (CPL)	CPL1	Being prepared for the unexpected and having back up plans	予期しない事態が起こったときのために、予備の計画を考えてある
	CPL2	Vision of where you want to go in life after your dual career	デュアル・キャリアを通して、自分がどうなりたいか見えている
	CPL3	Being curious to explore career plans outside elite sport	競技をやること以外に、自分のキャリアを探すことに興味がある
	CPL4	Ability to be flexible and change plans if necessary	必要に応じて、計画を柔軟に変更できる
	CPL5	Having knowledge about your career options in study and sport	学業とスポーツに関して、自分のキャリアにどんな選択肢があるのか知っている
Emotional awareness (EMA)	EMA1	Assertiveness (being self-assured and acting with confidence)	自信を持っており、確信を持って行動できる
	EMA2	Ability to cope with stress in sport and study	スポーツと学業のストレスに対処できる
	EMA3	Belief in your own ability to overcome the challenges in sport and study	自分はスポーツと学業において直面する困難を克服できると信じている
	EMA4	Ability to regulate emotions in different situations	どのような状況でも感情をコントロールできる
	EMA5	Ability to use setbacks in sport and/or study as a positive stimulus	スポーツと学業で挫折してもその後の糧にできる
	EMA6	Ability to focus on here and now, without being distracted	気を散らすことなく、今ここに集中できる
	EMA7	Being patient about the progression of your sport and study career	スポーツと学業のキャリアを、忍耐強く前進させることができる
Social intelligence & adaptability (SIA)	SIA1	Eagerness to listen and learn from others and past experiences	他人の経験談を聞きたい、過去の経験から学びたいと強く思っている
	SIA2	Asking advice to the right people at the right time	適切な時期に適切な人にアドバスを求めることができる
	SIA3	Ability to collaborate with support staff in study and sport	スポーツと学業において、支援してくれる人と良い関係を作れる
	SIA4	Ability to make social contacts with peers in study and sport	学業とスポーツのことで友人に相談できる
	SIA5	Ability to maintain relations with important others	他者と良い関係を維持できる
	SIA6	Understanding the importance of rest and recuperation	休むことや回復に努めることの重要性をよくわかっている
	SIA7	Ability to resolve conflicts	葛藤を解決できる

The DCCQ-A assesses two dimensions: the perceived importance of dual-career competencies and the extent to which individuals believe they possess these competencies. Participants responded to two sets of instructions: one asking “how important is this competence for you to successfully combine sport and study?”, and the other asking the degree to which they feel they currently possess those competencies, “to what extent do you possess this competence?”. The responses to the items on importance were rated on a 5-point Likert scale ranging from 1 (“Unimportant”) to 5 (“Very important”), while possession was assessed on a parallel 5-point scale ranging from 1 (“Very poor”) to 5 (“Very strong”). Cutoff values (≥4.0 = “important/possessed,” ≥3.0 = “somewhat important/possessed”) followed conventions from previous DCCQ-A research (16, 23), where responses of 4–5 indicate strong agreement and 3 reflects moderate endorsement.

Each of the 29 items was assigned to one of the four competency domains: dual career management (DCM), career planning (CPL), emotional awareness (EMA), and social intelligence and adaptability (SIA). The average scores were calculated by summing the item scores within each factor and then dividing the total by the number of items. A mean score above 4.0 was interpreted as indicating that the competency is “important” or “possessed,” while a score above 3.0 was considered “somewhat important” or “somewhat possessed.”

The translation process followed Brislin's back-translation method (28). Two bilingual experts independently translated the items, and two different bilingual translators conducted blind back-translations. Discrepancies were resolved through a panel of five experts in sports science, psychology, and higher education. Cultural adaptation was applied to ensure contextual relevance for Japanese student-athletes. A pilot test with 15 student-athletes was conducted to confirm clarity and comprehension, leading to minor wording adjustments (25).

### 2.3 Data analysis

To assess content validity, we calculated the Content Validity Coefficient (CVC) proposed by Hernández-Nieto (28). This evaluation involved five expert reviewers (one female and four males), including two specialists in sports management, two in higher education, and one in sports psychology, as well as ten elite student-athletes (five females and five males, *M* = 21.83, *SD* = 1.33) who competed in track and field (*n* = 5), swimming (*n* = 3), and gymnastics (*n* = 2). Each participant independently rated the relevance of each item on a 5-point scale ranging from 1 (“Not relevant at all”) to 5 (“Highly relevant”), after which item-level and overall adjusted CVC values were computed. The CVC for each item was calculated using the formula:

$$\mathrm{CVC}c=\frac{\sum x_{i}}{N\times k}$$

where *x_i_* represents the score assigned by each evaluator, *N* is the total number of evaluators (experts and athletes combined), and *k* is the maximum possible score on the scale. To adjust for chance agreement, the adjusted CVC was computed as:

$$\mathrm{CVC}aj=\mathrm{CVC}c-\frac{1}{N\times k}$$

To examine item discrimination, we conducted a Good–Poor (G–P) analysis by comparing the mean item scores between the top and bottom 25% of respondents based on the total DCCQ-AJ scores. The factorial structure of the DCCQ-AJ was assessed using confirmatory factor analysis (CFA), adopting the same four-factor model as the original. Model fit was evaluated using the root mean square error of approximation (RMSEA), comparative fit index (CFI) (29), and Tucker–Lewis index (TLI) (30). Model fit was considered acceptable if the RMSEA was below 0.08 and both CFI and TLI exceeded 0.90, as recommended by Hu and Bentler (31) and Marsh et al. (32). The internal consistency reliability was assessed by calculating Cronbach's alpha (33) for each competency factor. Following the validation process, we calculated descriptive statistics (means and standard deviations) for both the perceived importance and possession levels across the four competency domains. Paired-sample *t*-tests were conducted to compare perceived importance and possession scores. In addition to *p*-values, effect sizes (Cohen's *d*) and 95% confidence intervals for the mean differences were calculated to assess the magnitude and precision of the effects. All analyses were performed using IBM SPSS Statistics 27.0 and AMOS 27.0.

## 3 Results

### 3.1 Reliability and validity of the DCCQ-AJ

To evaluate the content validity of the DCCQ-AJ, we calculated the Content Validity Coefficient (CVC) in accordance with the method proposed by Hernández-Nieto (28). For the scale measuring the perceived importance of competencies, item-level CVCs ranged from 0.91 to 0.98, and the overall adjusted CVC was 0.81. For the scale measuring perceived possession, item-level CVCs ranged from 0.90 to 0.97, and the overall adjusted CVC was 0.82. These results indicated that both scales demonstrated strong content validity. To assess item discrimination, a G–P analysis was conducted. For all 29 items on both importance and possession scales, participants in the high-scoring group scored significantly higher than those in the low-scoring group (*p* < .001). Confirmatory factor analysis (CFA) revealed that the model fit indices for the importance scale were CFI = 0.96, TLI = 0.95, and RMSEA = 0.05. For the possession scale, the indices were as follows: CFI = 0.93, TLI = 0.91, and RMSEA = 0.05. Factor loadings ranged from 0.56 to 0.79 for importance items and 0.47 to 0.77 for possession items. Table 2 presents the means, standard deviations, and factor loadings for each item. To examine the internal consistency, Cronbach's alpha coefficients were calculated for each competency factor. The alpha coefficients for the importance scale ranged from 0.86 to 0.91, while those for the possession scale ranged from 0.76 to 0.86, indicating acceptable to excellent internal consistency (Table 2).

**Table 2:** Psychometric properties of the DCCQ-AJ: factor loadings and internal consistency.

Factors	items	Importance				Possession			
		*M*	*SD*	Loadings (*λ*)	*α*	*M*	*SD*	Loadings (λ)	*α*
Dual career management (DCM)	DCM1	4.03	0.88	0.75	0.91	3.78	0.95	0.60	0.86
	DCM2	4.05	0.93	0.72		3.67	1.05	0.58	
	DCM3	3.97	0.94	0.71		3.55	1.04	0.56	
	DCM4	3.96	0.90	0.68		3.50	1.04	0.55	
	DCM5	4.03	0.92	0.73		3.92	0.92	0.62	
	DCM6	3.84	0.97	0.56		3.72	0.97	0.57	
	DCM7	3.97	0.91	0.75		3.80	0.92	0.73	
	DCM8	3.96	0.95	0.67		3.71	0.97	0.64	
	DCM9	4.02	0.88	0.76		3.82	0.93	0.70	
	DCM10	3.96	0.94	0.68		3.68	0.97	0.60	
Career planning (CPL)	CPL1	3.89	0.96	0.72	0.86	3.45	1.05	0.61	0.76
	CPL2	3.88	0.98	0.75		3.46	1.01	0.68	
	CPL3	4.01	0.90	0.69		3.76	0.95	0.64	
	CPL4	4.04	0.93	0.79		3.83	0.89	0.70	
	CPL5	3.94	0.94	0.79		3.63	0.97	0.72	
Emotional awareness (EMA)	EMA1	3.95	0.96	0.74	0.90	3.59	1.05	0.68	0.84
	EMA2	4.06	0.90	0.71		3.62	0.97	0.61	
	EMA3	3.99	0.90	0.72		3.66	0.95	0.69	
	EMA4	4.05	0.92	0.74		3.68	0.98	0.53	
	EMA5	4.05	0.90	0.75		3.94	0.89	0.71	
	EMA6	4.03	0.91	0.76		3.75	0.95	0.68	
	EMA7	4.02	0.90	0.74		3.85	0.89	0.76	
Social intelligence & adaptability (SIA)	SIA1	4.01	0.92	0.66	0.88	3.97	0.91	0.58	0.80
	SIA2	4.02	0.93	0.77		3.73	0.93	0.65	
	SIA3	4.13	0.90	0.73		4.12	0.89	0.67	
	SIA4	4.03	0.96	0.70		3.91	1.02	0.56	
	SIA5	4.14	0.91	0.74		4.15	0.85	0.67	
	SIA6	4.26	0.88	0.61		4.34	0.88	0.47	
	SIA7	3.98	0.91	0.75		3.69	0.89	0.69	

### 3.2 Perceived importance and possession of dual career competencies

**Figure 1:**
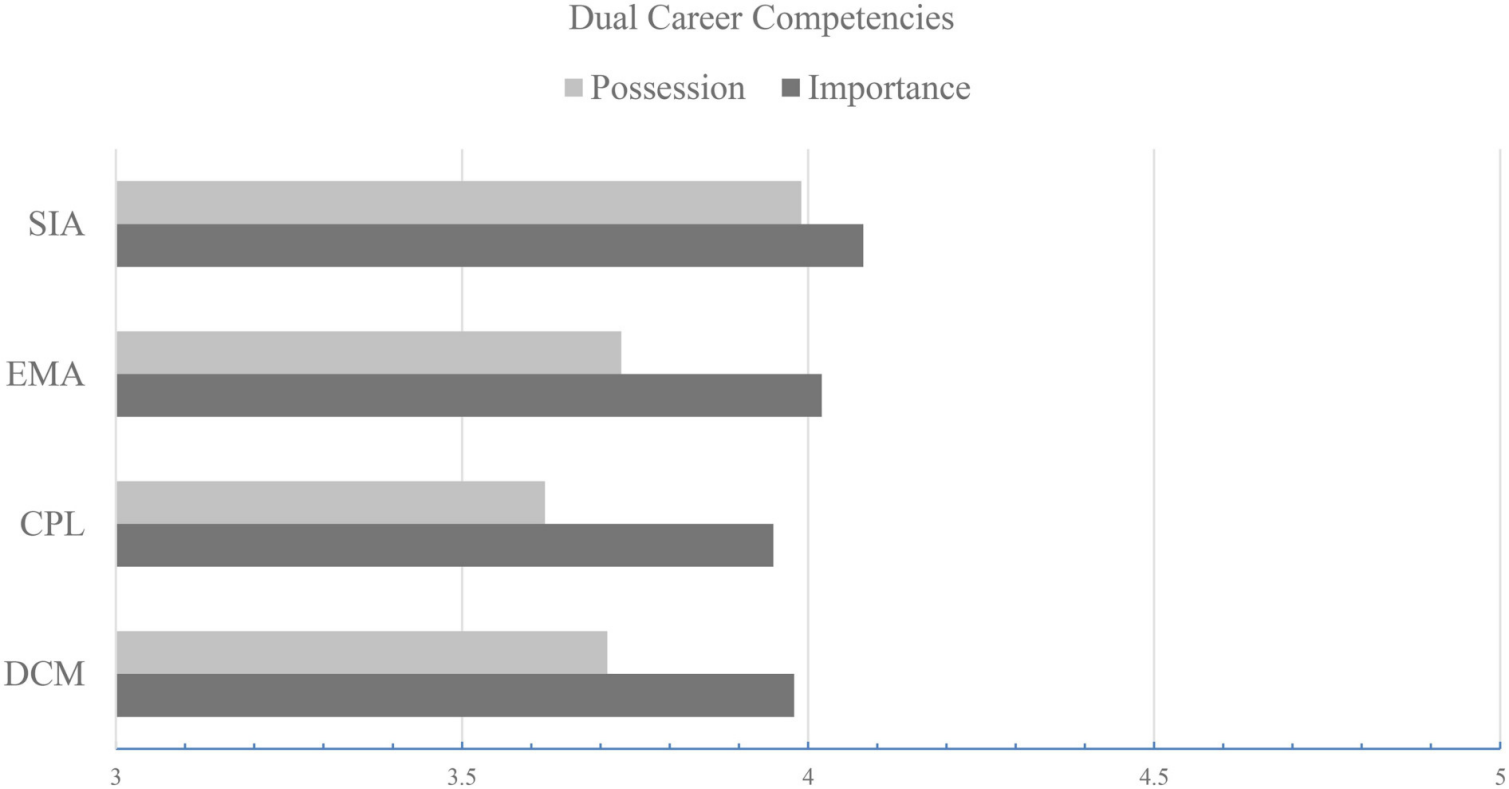
Comparison of mean perceived importance and possession across the four dual-career competency domains.

To examine the differences between perceived importance and perceived possession across each competency domain, paired-sample *t*-tests were conducted. Statistically significant differences were found in all four domains (*p* < .001), with mean importance scores consistently higher than possession scores. The magnitude of these differences ranged from small to moderate, as indicated by the corresponding effect sizes (Cohen's *d* = 0.17–0.47). In addition, the 95% confidence intervals for the mean differences did not include zero in any domain, confirming the robustness of the observed effects. These results suggest that student-athletes tend to place greater value on dual-career competencies than they perceive themselves as currently possessing. A summary of these comparisons, including confidence intervals and effect sizes, is presented in Table 3. To facilitate visual interpretation, Figure 1 presents a bar plot comparing the mean scores of perceived importance and perceived possession across the four competency domains. This graphical representation complements the statistical results reported in Table 3 and highlights the consistent gaps observed between the two dimensions.

**Table 3:** Comparison of perceived importance and possession of dual career competencies.

Competency factors	Importance *M* (*SD*)	Possession *M* (*SD*)	Difference *M* (*SD*)	*t*	*p*	95% CI of difference	Cohen's *d*
DC management (DCM)	3.98 (0.68)	3.71 (0.65)	0.26 (0.57)	14.93	***	[0.23, 0.31]	0.47
Career planning (CPL)	3.95 (0.76)	3.62 (0.71)	0.33 (0.72)	14.45	***	[0.29, 0.38]	0.46
Emotional awareness (EMA)	4.02 (0.72)	3.73 (0.69)	0.29 (0.69)	15.01	***	[0.26, 0.34]	0.47
Social intelligence & adaptability (SIA)	4.08 (0.69)	3.99 (0.62)	0.09 (0.56)	5.38	***	[0.06, 0.13]	0.17

*** *p* < .001. Values of Cohen's d are shown as absolute effect sizes (direction omitted for interpretability).

## 4 Discussion

This study aimed to validate and apply the DCCQ-AJ, which retains the same item count and factor structure as the widely used European original (16), to assess the current state of dual career competencies among Japanese student-athletes. Content validity analysis showed high item-level CVC values across both the importance and possession scales, with overall adjusted CVCs exceeding 0.80. These results indicate that the DCCQ-AJ has a strong conceptual alignment with the competencies it intends to measure (28). Notably, the high ratings of both researchers and student-athletes suggest that the scale is not only theoretically sound but also practical and easy to understand in real-world contexts. The G–P analysis revealed that all 29 items discriminated significantly between the high- and low-scoring groups (*p* < .001), confirming the discriminative power of each item. In addition, Cronbach's alpha coefficients for all subscales exceeded the standard threshold of 0.70 (33), indicating good internal consistency. Confirmatory factor analyses demonstrated good model fit for both the perceived importance and possession dimensions, supporting the factor validity of the DCCQ-AJ and confirming its structural equivalence with the original instrument. Unlike a previous pilot study by our team (25) that suffered from sample limitations, the current study employed a broader and more diverse sample, allowing for more robust psychometric validation. Establishing cross-cultural equivalence is essential to develop a Japanese version of the DCCQ-A. Previous studies have emphasized the importance of verifying measurement equivalence when adapting psychological scales for international comparison (34). Our findings suggest that the DCCQ-AJ demonstrates conceptual and functional equivalence to the original version, making it a valuable tool for both domestic and cross-national studies. It may also help identify the context-specific challenges faced by Japanese student-athletes in pursuing dual careers. Regarding the perceived importance of competencies, most items exceeded a mean score of 4.0, and all surpassed 3.0. These results indicate that Japanese student-athletes recognize these competencies as at least “somewhat important” to “important” for dual career development. This aligns with the findings of European studies that utilized the DCCQ-A (16, 23, 24), affirming that student-athletes in Japan, like their European counterparts, view these competencies as critical. Interestingly, while previous surveys in Japan reported a low awareness of the dual-career concept (13), the present study suggests that student-athletes understand and acknowledge the importance of specific competencies when presented concretely. This implies that raising awareness of dual careers may benefit from tools such as the DCCQ-AJ, making the concept more tangible. Coaches and administrators should support student-athletes in understanding both the general concept and specific skills required for successful dual-career development. For perceived possession, only three items “Ability to collaborate with support staff in study and sport,” “Ability to maintain relations with important others,” and “Understanding the importance of rest and recuperation” had mean scores above 4.0, suggesting relatively higher self-confidence in these areas. Most other items hovered just above 3.0, indicating that many student-athletes feel they possess these competencies only “to some extent.” These trends mirror the findings of earlier studies (16, 23, 24), reinforce the need for competency-building support.

Paired *t*-tests revealed significant discrepancies between perceived importance and possession across all four domains; this suggests that student-athletes are aware of the importance of these competencies but do not feel fully equipped to embody them. In addition to statistical significance, the magnitude of these differences ranged from small to moderate, as indicated by effect sizes, and the 95% confidence intervals for mean differences did not include zero, confirming the robustness of the observed differences. Linnér et al. (23) observed similar findings among Swedish student-athletes, which led to the development of national guidelines for dual-career support based on DCCQ-A data. Japan could likewise consider leveraging the DCCQ-AJ to inform the creation of evidence-based development guidelines led by institutions such as the UNIVAS. A study by Pérez-Rivases et al. (35) in Spain also reported significant gaps between perceived importance and possession of dual career competencies, particularly in DCM and EMA, suggesting that these domains require the most support. By visualizing the gaps between perceived importance and possession, the DCCQ-A can help to identify concrete areas for improvement in dual-career training programs (16). Japan should use the DCCQ-AJ to investigate how to optimize support environments and educational programs for student-athletes. Notably, the domains with the largest gaps such as EMA and DCM may reflect the limited availability of formal psychological and career guidance services within Japanese university sport settings. Unlike several European countries where structured dual-career pathways and psychosocial support systems are well established (36), Japan's support infrastructure tends to be informal and highly dependent on institutional context (37). Furthermore, as Noguchi et al. (38) point out, many Japanese student-athletes hesitate to seek professional or interpersonal support, with nearly half reporting that they have no one to talk to about personal concerns. This reluctance is closely tied to cultural norms that value perseverance, emotional restraint, and interpersonal harmony, which can suppress self-expression and proactive help-seeking. These tendencies may limit the development or utilization of competencies such as emotional regulation and autonomous decision-making, even when athletes recognize their importance. Additionally, alternative admission systems such as athletic recommendation or admissions office pathways that do not necessarily require academic testing have proliferated in lower-ranked universities (4, 5), potentially resulting in student-athletes entering higher education without adequate access to dual-career guidance or structured developmental support. Consequently, a subset of student-athletes enter higher education without clear academic goals or career plans (5). Most athletes do not receive substantial support such as scholarships or tutoring and are classified as sub-elite athletes who must finance their education independently (37). These sub-elite athletes exhibit lower motivation and commitment to both sports and academia (4). Thus, dual career strategies should not be limited to elite athletes but should also encompass a broader population of sub-elite student-athletes. Taken together, the current findings align with the broader literature from Europe in terms of competency trends (16, 23, 35), but the magnitude and distribution of perceived possession may be shaped by Japan's unique cultural and institutional landscape. Although the present study treated Japanese student-athletes as a single group for analytical purposes, the sample comprised individuals from a wide range of sports, competitive levels, and university systems. These forms of heterogeneity may influence both the opportunities for support and the development of dual-career competencies. For instance, athletes in team-based or Olympic disciplines may benefit from more structured guidance and resources than those in non-Olympic or emerging sports. Likewise, students enrolled at higher-resourced private universities may have access to different forms of academic or career support compared to those in regional or lower-ranked institutions. Recognizing this diversity is important for interpreting the current findings and underscores the need to examine subgroup differences in future research. Future studies could adopt a comparative cross-cultural design to further investigate contextual variables such as national policies, cultural values, and university support systems moderate dual-career development. Since Japan's athlete population is more diverse than that of many Western countries, where support is often limited to internationally competitive athletes, domestic policy must be tailored accordingly. The DCCQ-AJ should be employed to develop competency-based dual-career programs customized for different athletic levels and educational backgrounds. This approach provides a more inclusive and effective support for Japan's unique student-athlete population.

Despite the strengths of this study (such as the large sample and robust validation process), several limitations should be noted. First, our analysis treated the sample as a single group; we did not examine competency differences between sports, competitive levels, or university systems. It remains possible that, for example, student-athletes in individual sports face different dual-career challenges than those in team sports, or that athletes at top-tier universities (with more support services) possess higher competency levels than those at institutions with fewer resources. Future research should stratify and compare such subgroups to determine how context influences dual-career competencies, as suggested by broader dual-career frameworks. Second, the study relied on self-reported measures of perceived importance and possession of competencies. Self-reporting can introduce biases (e.g., social desirability or differences in self-awareness), meaning that some student-athletes might overestimate or underestimate their true competence levels. Triangulating these findings with qualitative interviews or coach evaluations could provide a more objective picture. Third, our cross-sectional design offers a snapshot of dual-career competencies but cannot capture how these competencies develop or change over time. Longitudinal studies would be valuable to track student-athletes throughout their university years, examining when and how improvements in competencies occur and whether they translate into better dual-career outcomes (such as academic performance, well-being, or smoother career transitions). Building on these limitations, we outline several avenues for future research. One priority is to investigate how different support environments impact dual-career development. For example, researchers could compare student-athletes who are part of formal dual-career support programs (or sports-specific academic tracks) with those who receive minimal support, to assess the effect on competency acquisition. Additionally, given that the DCCQ-AJ is equivalent in structure to the original European DCCQ-A, cross-cultural studies are now feasible: future work can benchmark Japanese student-athletes' competencies against those in other countries to identify unique challenges or best practices. Another promising direction is intervention-based research. The significant gaps we identified between perceived importance and possession of competencies highlight areas for improvement; experimental studies or program evaluations could be conducted where specific interventions (e.g., time-management workshops, career planning seminars, or mental health and resilience training) are implemented and student-athletes' DCCQ-AJ scores are tracked pre- and post-intervention. Such studies would test whether targeted support can effectively close the competency gaps and improve dual-career readiness. In summary, acknowledging the diversity within the student-athlete population and addressing the noted limitations will not only refine our understanding of dual-career competencies but also guide the development of more nuanced, evidence-based support systems. Through continued research in these areas, we can work toward ensuring that all student-athletes across various sports, competitive levels, and institutions are equipped to succeed in both their athletic and academic pursuits.

## 5 Conclusion

This study validated the reliability and validity of the DCCQ-AJ, a web-based adaptation of the widely used and multilingual DCCQ-A. The DCCQ-AJ preserved its original structure and item composition and demonstrated sufficient psychometric properties, enabling its use in evaluating dual-career competencies among student-athletes in Japan. The findings revealed that while student-athletes recognized the importance of dual-career competencies, their perceived possession of these competencies lagged behind. This discrepancy highlights the need for systematic support programs tailored to develop these competencies. The DCCQ-AJ is a standardized and scalable tool for monitoring and visualizing student-athlete competencies nationwide. Given its online format, the DCCQ-AJ is well suited for large-scale implementation and longitudinal monitoring. It offers coaches and administrators a foundation for identifying athletes' needs and designing individualized support strategies. Its utility as an evaluation tool also makes it valuable for coaches to assess and support dual-career development within teams.

## Data Availability

The raw data supporting the conclusions of this article will be made available by the authors, without undue reservation.
